# Pharmacogenetics of *ABCB1* , *CDA* , *DCK* , *GSTT1* , *GSTM1* and outcomes in a cohort of pediatric acute myeloid leukemia patients from Colombia

**DOI:** 10.1002/cnr2.1744

**Published:** 2022-10-31

**Authors:** Luz K. Yunis, Adriana Linares‐Ballesteros, Nelson Aponte, Gisela Barros, Johnny García, Laura Niño, Gloria Uribe, Edna Quintero, Juan J. Yunis

**Affiliations:** ^1^ Grupo de Patología Molecular Universidad Nacional de Colombia Bogotá Colombia; ^2^ Servicios Médicos Yunis Turbay y Cía S.A.S Instituto de Genética Bogotá Colombia; ^3^ Unidad de Oncología/Hematología Pediátrica HOMI Fundación Hospital Pediátrico La Misericordia Bogotá Colombia; ^4^ Grupo de Oncohematología Pediátrica Universidad Nacional de Colombia‐HOMI Fundación Hospital Pediátrico La Misericordia Bogotá Colombia; ^5^ Unidad de Patología HOMI Fundación Hospital Pediátrico La Misericordia Bogotá Colombia; ^6^ Departamento de Patología, Facultad de Medicina e Instituto de Genética Universidad Nacional de Colombia Bogotá Colombia

**Keywords:** *ABCB1*, acute myeloid leukemia, anthracyclines, *CDA*, Colombia, cytarabine, *DCK*, *GSTM1*, *GSTT1*, SNV

## Abstract

**Background and Aim:**

Different studies have shown pharmacogenetic variants related to drug toxicity in acute myeloid leukemia (AML) patients. Our aim was to identify the association between *ABCB1, CDA*, *DCK*, *GSTT1*, and *GSTM1* variants with clinical outcomes and toxicity in pediatric patients with AML.

**Methods:**

Fifty‐one confirmed de novo AML pediatric patients were included. A SNaPshot™ assay and conventional PCR were used to evaluate *ABCB1, CDA*, *DCK*, *GSTT1*, and *GSTM1* variants. Clinical outcomes and toxicity associations were evaluated using odds ratios and Chi‐square analysis.

**Results:**

Patients carrying *ABCB1* (1236C > T, rs1128503) GG genotype in had a 6.8 OR (CI 95% 1.08–42.73, *p* = .044) for cardiotoxicity as compared to patients carrying either AA or GA genotypes 0.14 OR (CI 95% 0.023–0.92, *p* = .044). For *ABCB1* (1236G > A rs1128503/2677C > A/T rs2032582/3435G > A rs1045642) AA/AA/AA combined genotypes had a strong association with death after HSTC OR 13.73 (CI 95% 1.94–97.17, *p* = .009). Combined genotypes GG/CC/GG with *CDA* (79A > C, rs2072671) CA genotype or *CDA* (‐451G > A, rs532545) CT genotype, had a 4.11 OR (CI 95% 2.32–725, *p* = .007) and 3.8 OR (CI 95% 2.23–6.47, *p* = .027) with MRD >0.1% after first chemotherapy cycle, respectively.

**Conclusion:**

Our results highlight the importance of pharmacogenetic analysis in pediatric AML, particularly in populations with a high degree of admixture, and might be useful as a future tool for patient stratification for treatment.

## 1 INTRODUCTION

Acute myeloid leukemia (AML) comprises a heterogenous group of diseases with dismal outcomes mainly in developing countries.^1^ Identification of genetic alterations is key for risk stratification, as it guides to appropriate treatment.^2^ Most patients with AML are classified as intermediate or unfavorable risk, usually leading to failure of induction therapy and early relapse after achieving complete remission.^3^ The identification of gene variants that participate in the processes of pharmacodynamics and pharmacokinetics (pharmacogenetics), have become relevant in the era of personalized medicine, in terms of the influence they exert on the response to treatment,^4^, ^5^, ^6^ and the incorporation of interindividual genetic differences for the design of more effective diagnostic and therapeutic strategies.^7^, ^8^, ^9^, ^10^, ^11^ Cytarabine (Cytosine arabinoside Ara‐c) and anthracyclines are routinely used in front‐line therapy for AML.^12^, ^13^, ^14^, ^15^ Resistance development to chemotherapy is a major obstacle in AML treatment and is responsible for relapses and increased toxicity in second‐line therapies.^16^ Ara‐C is a pyrimidine analog which is converted into ara‐CMP by deoxycytidine kinase (*DCK*), later on is converted to ara‐CDP and into ara‐CTP by cytidine/uridine monophosphate kinase 1 (*CMPK1*) and nucleoside diphosphate kinase 1 (NME1), respectively. Ara‐CTP is a competitor of deoxycytidine 5′‐triphosphate that acts by inhibiting DNA synthesis. Cytidine deaminase (*CDA*) converts ara‐C to the inactive metabolite uracil arabinoside (ara‐U), which limits the amount of ara‐C to be converted to ara‐CTP.^6^, ^17^, ^18^, ^19^ *ABCB1*, also known as multi‐drug resistance protein 1 (MDRP1), is 1 of 49 putative members in the superfamily of human adenosine triphosphate (ATP)‐binding cassette (ABC) transporters that encode transporter and channel proteins that function as efflux pumps,^20^, ^21^ codifies a P‐glycoprotein efflux transporter involved in mediating resistance to several drugs, multidrug resistance phenotype, in cancer^6^, ^18^ (Figure S1). Different studies have shown that single nucleotide variants (SNV) in *ABCB1, CDA, DCK, GSTT1*, and *GSTM1* genes are related to drug toxicity in patients with AML. Two main SNV in the deoxycytidine kinase (*DCK*) gene (‐360C > T, rs377182313) and (‐201C > A, rs2306744), have been described associated to pharmacogenetic responses.^22^ Whether *DCK* mutations make AML cells resistant to cytarabine is controversial.^23^ Cytidine deaminase (*CDA*) irreversibly deaminates cytarabine, its overexpression results in Ara‐C resistance, while decreased expression is associated with toxicity. Two SNV in *CDA* gene have been found associated with pharmacogenetic responses, (79A > C, rs2072671) and (‐451C > T, rs532545).^24^, ^25^ For *ABCB1*, three SNV have been associated to pharmacogenetic responses, *ABCB1* (1236G > A, rs1128503), *ABCB1* (2677C > A/T, rs2032582) and *ABCB1* (3435G > A, rs1045642).^26^, ^27^, ^28^

Several antineoplastic drugs are metabolized by glutathione S‐transferase (GST) which catalyzes the conjugation of reduced glutathione to electrophilic centers of platinum drugs, anthracyclines, vinca alkaloids, cyclophosphamide, and epipodophylotoxins.^29^

The *GSTT1* gene encodes the phase II metabolizing enzyme glutathione s‐transferase theta and *GSTM1* encodes glutathione S‐transferase mu 1.^30^ The most studied variants of *GSTT1* and *GSTM1* are the null variant, which results from the complete or partial deletion of these genes. It has been suggested that individuals lacking *GSTT1* and or *GSTM1* have an impaired ability to detoxify environmental xenobiotics and are thus at elevated risk for cellular damage and resultant cancer.

In the present study we analyzed 6 SNV in *ABCB1* gene (3435C > T, rs1045642), (1236G > A, rs1128503), (2677G > T/A, rs2032582); *CDA* gene (79A > C, rs2072671), (‐451C > T, rs532545); *DCK* gene (‐201C > A, rs2306744), and the presence or absence of the *GSTT1* and *GSTM1* null alleles and their correlation with clinical outcomes and toxicity in a cohort of pediatric patients with AML from two pediatric cancer treatment centers in Colombia.

## 2 MATERIALS AND METHODS

### 2.1 Patients

Descriptive observational cohort study, 51 pediatric patients with a confirmed diagnosis of de novo AML (non‐promyelocitic) by convenience were included, with prior informed consent between March 2015 and June 2021, from HOMI Fundación Hospital Pediátrico La Misericordia and Clínica Infantil Colsubsidio Bogotá D.C., Colombia. Patients with Down syndrome or secondary AML were not included. This project was approved by the ethics committee of each institution, CEI 125‐18 and 243‐1, respectively, and the ethics committee of Universidad Nacional de Colombia (007‐091‐18). Patients received two cycles of induction chemotherapy including cytarabine 100 mg/m^2^/day for 7 days and daunorubycin 60 mg/m^2^/day for 3 days “7 × 3 cycle.” Consolidation chemotherapy was based on cytarabine high dose 3 mg/m^3^/day for 3 days, 2 or 3 cycles maximum. Minimal residual disease (MRD) evaluation was made based on multiparametric flow cytometry on bone marrow samples. Residual disease was detected using the leukemia‐associated immunophenotype at diagnosis and at follow‐up samples. A cutoff value of 0.1% was used as the threshold to distinguish MRD‐positive from MRD‐negative patients, acquiring around 2.5 million events (excluding all CD45‐negative cells and debris).^31^

### 2.2 Pharmacogenetic testing

We used a SNaPShot™ (Thermo Fisher Scientific) panel to simultaneously test for SNV (3435G > A, rs1045642), (1236C > T, rs1128503), (2677C > A/T, rs2032582) in *ABCB1* gene; (79A > C, rs2072671), (‐451C > T, rs532545) *CDA* gene, and separately, (‐201C > A, rs2306744) in *DCK* gene.

Primers and probes used for the SNaPshot™ assay are listed in Table 1. Primers were designed in order to perform a multiplex PCR reaction using Qiagen 2X PCR multiplex master mix (Qiagen). PCR amplification conditions were an initial denaturing step at 95°C for 15 min, followed by 32 cycles of denaturation at 94°C for 30 s, annealing at 60°C for 90 s followed by extension at 72°C for 60 s, with a final extension step at 72°C for 10 min. For *DCK* rs2306744, PCR amplification as described previously.^32^ Amplified products were purified according to the SNaPshot™ protocol, following manufacturers' recommendations. An aliquot of the purified sample was hybridized with probes designed to align to the specified genetic variant with an additional tail of nonhuman DNA sequences to obtain better separation of each variant.^33^ The purified PCR products were analyzed in a ABI3500 Genetic Analyzer using Liz120 as sizing standard and analysis software GeneMapper 4.2 (Applied Biosystems, Thermo Fisher Scientific).

**TABLE 1:** Primers and probes for SNaPshot™ for *ABCB1*, *DCK*, *CDA*, *GSTT1*, and *GSTM1*

Gene	SNV	rs	Primers/probes 5′‐3′	SNaPshot amplicon (bp)
*DCK*	‐201C > T	rs2306744	F‐CTGCAGGTGACGCCCTCT	469
			R‐GGGTGGCCATTCCTTAGTCT	
			P‐ACGTCGTGAAAGTCTGACAACTGGCGGGCCTGCGG	35
*CDA*	79A > C	rs2072671	F‐AGGAGCTGCAATCGTGTCT	198
			R‐AGGAAAGTGACTGTAGGGGC	
			P‐TGCCACGTCGTGAAAGTCTGACAACTCCCAGGAGGCCAAG	40
	‐451G > A	rs532545	F‐GCCTCAGCCTCCTAAAGTGA	264
			R‐CAAAGGTCCAAGCTCCAAGG	
			P‐ACGTCGTGAAAGTCTGACAACTGGCGGGCCTGCGG	48
*ABCB1*	3435G > A	rs1045642	F‐GAAGAGAGACTTACATTAGGCAGTG	171
			R‐ACCTGGGCATCGTGTCC	
			P‐TCTGACAATCCTTTGCTGCCCTCAC	25
	1236G > A	rs1128503	F‐CTTCCCACAGCCACTGTTTC	124
			R‐CCTGTGTCTGTGAATTGCCTT	
			P‐GGTGCCACGTCGTGAAAGTCTGACAACTGCACCTTCAGGTTCAG	44
	2677C > A/T	rs2032582	P‐TGAAAAAGATTGCTTTGAGGAATGG	221
			R‐CCATCATTGCAATAGCAGGAGT	
			P‐ACGTCGTGAAAGTCTGACAACTGGCGGGCCTGCGG	28
*GSTM1*	Null		F‐GGAACTCCCTGAAAAGCTAAAGC	220
			R‐CTTGGGCTCAAATATACGGTGG	
*GSTT1*	Null		F‐GCCTTCCTTACTGGTCCTCA	383
			R‐AGAATGACCTCATGGGCCTC	

Abbreviations: F, forward; P, probe; R, reverse; SNV, single nucleotide variant.

The *GSTT1* and *GSTM1* deletions were analyzed by conventional PCR (Table 1). Each reaction also contained a control gene *ABCB1* (rs1045642) for amplification control. PCR amplification conditions were an initial denaturing step at 95°C for 3 min, followed by 32 cycles of denaturation at 94°C for 30 s, annealing at 60°C for 30 s followed by extension at 72°C for 60 s, with a final extension step at 72°C for 10 min. The PCR products were resolved by 2% Nusieve gel electrophoresis in 1X TBE buffer.

### 2.3 Genotype, allele frequencies, and Hardy Weinberg equilibrium

Genotype and allele frequencies were determined by direct counting method. Hardy Weinberg equilibrium was calculated based on observed and expected genotype frequencies. Genotypes for each gene variant obtained for each sample tested are listed in Table S1.

### 2.4 Statistical analysis

Quantitative variables were reported as means or medians with dispersion measures given in standard deviation and ranges, according to the nature and distribution of the variables, based on Shapiro Wilks normality test to establish the use of parametric tests or non‐parametric. We evaluated the association of genotypes from each gene variant, as well as the co‐occurrence of genotypic variants in the *ABCB1, DCK*, and *CDA* with clinical outcomes and toxicity using Chi‐square. Qualitative variables were analyzed with Pearson's Chi‐square test and Fisher's exact test. Statistical analysis was performed using the Statistical Package for the Social Sciences (SPSS) for Windows, version 25.0. A *p*‐value <.05 was considered significant. Logistic regression analysis was performed to analyze the relationship of the different variables with outcomes, MRD, relapse, event‐free survival, and overall survival.

### 2.5 Outcomes and definitions

Organ toxicity was evaluated according to the National Cancer Institute Common Terminology Criteria for Adverse Events (CTCAE) Version 5.0,^34^ grading Scales 3–4 included: colitis, mucositis, cardiotoxicity, transaminitis, and aspergilosis (Table S2). In addition, we also evaluated the efficacy of induction therapy, after 2 cycles 7 × 3, with complete remission (less than 5% of morphological blast count in bone marrow smear and hematological recovery in peripheral blood with platelet count >50 000/μl, >1000/μl leukocytes and absolute neutrophil count >500/μl) and induction failure (>5% of morphological blast count in bone marrow smear and without hematological recovery in peripheral blood counts), relapse and HSCT related toxicity. Other outcomes were overall survival, defined as the time between diagnosis and last contact alive or dead; event‐free survival was defined as the time between diagnosis and death, induction failure, relapse, abandonment, change of treatment institution, or last contact alive.

## 3 RESULTS

Fifty‐one patients were included, demographic and clinical characteristics are shown in Table 3. Thirty patients (58%) were male, M:F ratio 1.4:1, the median age was 10 years (0.15–18 years), median leukocytes at diagnosis was 25 580 × 10^9^/L (1.190–1.896.000), IQR 87020, and CNS involvement in 16 patients (31%). One patient died before starting treatment. Thirty‐three (65%) patients required HSCT. Demographic and clinical data are shown in Table 2.

**TABLE 2:** Demographic and clinical characteristics of pediatric AML patients

Diagnosis		*n* (%)
	WBC	
	<20 × 10^9^/L	24 (47)
	20 a 100 × 10^9^/L	15 (29.4)
	>100 × 10^9^/L	12 (23.5)
	Blasts (%)	
	CNS involvement	16 (31.3)
	Gender	
	Female	21 (41.1)
	Male	30 (58.8)
Cytogenetics	t(8;21)	7 (15.2)
	Inv16	5 (10.8)
	*KMT2A* rearrangements	9 (19.5)
	Complex karyotype	2 (4.3)
	Other	10 (21.7)
Molecular	*FLT3*	14 (27.5)
	ITD	9 (17.6)
	TKD	5 (9.8)
	*NRAS*	11 (21.6)
	*KRAS*	7 (13.7)
	*WT1*	6 (11.8)
	*KIT*	6 (11.8)
Treatment	Induction	46 (92)
	Consolidation	36 (81.8)
	HSCT	33 (64.7)
	Autologous	9 (17.6)
	Umbilical cord blood	14 (27.5)
	Haploidentical	10 (19.6)
	Treatment related toxicity	49 (96)
	Mucositis	25 (49)
	Colitis	24 (47)
	Transaminitis	23 (45)
	Cardiotoxicity	7 (13.7)
	Aspergilosis	2 (3.9)
	HSCT related	21 (41.1)
	GvHD	9 (17.6)
Treatment response	MRD after First cycle 7 × 3	
	<0.1%	13 (25.5)
	0.1%–10%	20 (39.2)
	>10%	8 (15.7)
	Not available	10 (19.6)
	MRD after second cycle 7 × 3 (end of induction)	
	<0.01%	28 (54.9)
	≥0.01%	14 (27.5)
	Not available	9 (17.6)
	Risk classification end of induction	
	Low risk	4 (7.8)
	Intermediate Risk	8 (15.6)
	High risk	34 (66.6)
	Not available	5 (9.8)
Events		
	Remission	18 (35.2)
	Relapse	14 (27.5)
	Failure at end of induction (>5% blasts morphological on bone marrow)	7 (13.7)
	Death during induction phase	3 (5.9)
	Death during treatment (in remission)	4 (7.8)
	Death before treatment	1 (1.9)
	Toxicity related death	4 (7.8)
	HSCT related death	9 (17.6)

Abbreviations: CNS, central nervous system; GvHD, graft versus host disease; HSCT, hematopoietic stem cell transplantation; MRD, measurable residual disease; WBC, white blood count.

Genotypes and allele frequencies for each of the gene variants analyzed for *ABCB1, CDA, DCK, GSTT1*, and *GSTM1* are shown in Table 3. All genotypes tested were found to be in Hardy–Weinberg equilibrium (data not shown).

**TABLE 3:** Genotypes and allele frequencies among pediatric AML patients

Gene	*ABCB1*	*ABCB1*	*ABCB1*	*CDA*	*CDA*	*DCK*	*GSTT1*	*GSTM1*
SNV	1236C > T	2677C > A/T	3435G > A	‐451C > T	79A > C	‐201C > A	−	−
rs	rs1128503	rs2032582	rs1045642	rs532545	rs2072671	rs2306744	−	−
**Genotype**								
GG	0.28		0.235			0.868		
GA	0.4		0.529			0.128		
AA	0.34	0.255	0.235		0.373	0.005		
AT		0						
CC		0.216		0.353				
CA		0.51			0.627			
CT		0.02		0.569				
TT				0.078				
+/+							0.577	
+/−							0.308	
−/+							0.038	
−/−							0.058	
**Allele**								
G	0.4712		0.5			0.93		
A	0.5288	0.51	0.5		0.686	0.07		
C		0.48		0.637	0.314			
T		0.01		0.363				
+							0.902	0.627
−							0.098	0.373

Several associations between genotypic variants and toxicity outcomes were found. First, we analyzed each genotypic variant independently and later on, genotypic associations within each gene or between genes. Odds ratios, CI 95%, and *p*‐values are shown in Table 4.

**TABLE 4:** Associations between *ABCB1*, *CDA,* and *DCK* genotypic variants and outcomes

Gene(s)	Genotype(s)	Toxicity	OR	CI 95%	*p*
*ABCB1*	1236GG	Cardiotoxicity consolidation	6.8	1.08–42.73	.044
*ABCB1*	3435GG	Transaminitis_first induction cycle	4.51	1.15–17.75	.032
*ABCB1*	3435AA	Relapse	0.69	0.56–0.85	.025
*CDA*	‐451CC	Colitis_first induction cycle	0.2	0.048–0.828	.019
*DCK*	‐201GA	Toxicity related to HSCT	0.088	0,08‐0,921	.037
*ABCB1*	1236AA + 1236GA	Cardiotoxicity_first induction cycle	0.14	0.023–0.92	.044
*ABCB1*	3435AA + 3435GA	Transaminitis_first induction cycle	0.22	0.05–0.87	.032
*ABCB1*	3435GG + 3435GA	Relapse	1.44	1.17–1.78	.025
*CDA*	‐451CC + 79AA	Mucositis_first induction cycle	4.84	1.28–18.25	.016
*CDA*	‐451CC + 79AA	Transaminitis_first induction cycle	7.45	1.89–29.34	.004
*CDA*	‐451CC + 79AA	Transaminitis consolidation	8	1.34–47.77	.022
*CDA*	‐451CC + 79AA	Transaminitis_overall	5.28	1.45–19.16	.010
*CDA*	‐451CT + 79CA	Mucositis_first induction cycle	0.27	0.083–0.878	.026
*CDA*	‐451CT + 79CA	Transaminitis_first induction cycle	0.136	0.032–0.583	.005
*CDA*	‐451CT + 79CA	Transaminitis consolidation	0.103	0.011–0.932	.025
*ABCB1*	1236GA + 2677CA + 3435GA	Aspergilosis_first induction cycle	0.107	0.013–0.909	.017
*ABCB1*	1236GA + 2677CA + 3435GA	Relapse	4.33	1.09–17.10	.037
*ABCB1*	1236AA + 2677AA + 3435AA	Death related to HSCT	13.73	1.94–97.17	.009
*ABCB1*	1236AA2677AA + 3435AA	Relapse	0.632	0.495–0.805	.040
*ABCB1 + CDA*	1236GG + 2677CC + 3435GG + 79CA	MRD≥0.1% day 15	4.11	2.32–725	.007
*ABCB1 + CDA*	1236GG + 2677CC + 3435GG + 79CA	MRD≥1% day 15	2.84	1.84‐4.41	.024
*CDA + ABCB1*	‐451CT + 1236GG + 2677CC + 3435GG	MRD≥0.1% day 15	3.8	2.23‐6.47	.027

*Note*: *ABCB1* gene: (3435G > A, rs1045642), (1236C > T, rs1128503), (2677C > A/T, rs2032582); *CDA* gene: (79A > C, rs2072671), (‐451C > T, rs532545); *DCK* gene: (‐201C > A, rs2306744).

Abbreviations: HSCT, Hematopoietic stem cell transplantation; MRD, measurable residual disease.

We found that patients carrying *ABCB1* (1236C > T, rs1128503) GG genotype in had a 6.8 OR (CI 95% 1.08–42.73, *p* = .044) for cardiotoxicity at the end of induction, compared to patients carrying either AA or GA genotypes 0.14 OR (CI 95% 0.023–0.92, *p* = .044). Patients carrying *ABCB1* (3435C > T, rs1045642) GG genotype had a 4.51 OR (CI 95% 1.15–17.75, *p* = .032) for transaminitis, as opposed to those carrying either AA or GA genotypes 0.22 OR (CI 95% 0.05–0.87, *p* = .032). Also, *ABCB1* (3435C > T, rs1045642) AA genotype was identified as a protective factor for relapse 0.69 OR (CI 95% 0.56–0.85, *p* = .025), compared to those patients with either GG or GA genotype 1.44 OR (CI 95% 1.17–1.78, *p* = .025).

For *ABCB1* (1236G > A rs1128503/2677C > A/T rs2032582/3435G > A rs1045642) AA/AA/AA combined genotypes, a strong association was found with death after HSTC OR 13.73 (CI 95% 1.94–97.17, *p* = .009). In addition, these genotypes were protective factors against relapse 0.632 OR (CI 95% 0.495–0.805, *p* = .040).

Measurable residual disease (MRD) >0.1% after first cycle of chemotherapy was associated with *ABCB1* (1236G > A rs1128503/2677C > A/T rs2032582/3435G > A rs1045642) genotypes GG/CC/GG in addition to *CDA* (79A > C, rs2072671) CA genotype with 4.11 OR (CI 95% 2.32–725, *p* = .007), and *CDA* (−451G > A rs532545) CT genotype also was associated with 3.8 OR (CI 95% 2.23–6.47, *p* = .027).

*ABCB1* (1236G > A rs1128503/2677C > A/T rs2032582/3435G > A rs1045642) genotypes GG/CC/GG in addition to *CDA* (79A > C, rs2072671) CA genotype, showed a risk association with MRD >0.1% after first chemotherapy cycle 4.11 OR (CI 95% 2.32–725, *p* = .007), and *CDA* (−451G > A rs532545) CT genotype also was associated with MRD >0.1% after first chemotherapy cycle 3.8 OR (CI 95% 2.23–6.47, *p* = .027).

Genotype GA in *DCK* (‐201C > A, rs2306744) is a protective factor to develop toxicity related to HSCT 0.8 OR (CI 95% 0.36–0.68, *p* = .046).

*CDA* (−451G > A, rs532545) genotype CC was found to be a protective factor for colitis 0.2 OR (CI 95% 0.048–0.828, *p* = .019) in our cohort. Combined genotypes for *CDA* (−451G > A rs532545) and (79A > C, rs2072671) CC/AA were associated with increased risk for mucositis and liver toxicity after the first 7 × 3 cycle and after consolidation, while genotypes CT/CA were a protective factor.

We did not find any association between *GSTT1* and *GSTM1* null alleles with clinical or toxicity events.

A logistic regression model was performed to evaluate the presence of independent predictors associated with relapse, finding a positive association for event‐free survival (relapse or death) and for overall survival with the presence of *ABCB1* 1236/2677/3435 GA/CA/GA 9.086 OR (CI 95% 1.669–49.466, *p* = .011). No associated predictors were found for overall survival or MRD.

## 4 DISCUSSION

Few studies have analyzed pharmacogenetic risk associations in pediatric AML patients. Most patients with AML are classified, using conventional cytogenetics, recurrent mutations, and response at the end of induction using morphological or MRD counts at different timelines, there are no prognostic genomic or molecular criteria routinely used to identify patients at risk of chemotherapy failure. Genetic background for genes involved in pharmacological response represents an additional factor in treatment response.^4^, ^5^, ^6^ Identification of pharmacogenetic determinants are potential predictive markers for treatment‐related adverse events and toxicity and in establishing differences in treatment schemes or intensification of therapy in post‐induction phase.^3^

Green et al, informed association between *ABCB1* (1236C > T, rs1128503), GG genotype with decreased survival when treated with cytarabine in AML patients as compared with AA + AG genotypes,^26^ in our cohort we found for *ABCB1* (1236C > T, rs1128503) GG genotype an increased risk for cardiotoxicity 6.8 OR (CI 95% 1.08–42.73, *p* = .044), while AA or GA genotype were a protective factor against cardiotoxicity OR 0.14 (CI 95% 0.023–0.92, *p* = .044). In the same study, *ABCB1* (2677C > A/T, rs2032582) CC genotype was associated with decreased survival when patients with AML were treated with cytarabine compared to genotypes AA + AC, we did not find any association between *ABCB1* (2677C > A/T rs2032582) CC genotype and survival or toxicity. *ABCB1* (3435G > A, rs1045642) GG genotype was associated with increased likelihood of complete remission when treated with cytarabine and idarubicin in AML patients as compared to AA + AG genotypes,^26^ other study showed for *ABCB1* (3435G > A, rs1045642) AA + AG genotypes were associated with increased overall survival when treated with cytarabine in AML patients as compared to genotype GG.^27^ In our patients for *ABCB1* (3435G > A, rs1045642) GG genotype we found an association with toxicity, and not with the response at the end of induction. While with AA genotype we have a protective factor for relapse 0.69 OR (CI 95% 0.56–0.85, *p* = .025) in contrast with the study mentioned previously.^26^ In a previous study, the *ABCB1* triple variant haplotype *ABCB1* (1236G > A rs1128503/2677C > A/T rs2032582/3435G > A rs1045642) TT/TT/TT was related to increased nephrotoxicity than other genotypes.^28^ For the combined genotypes *ABCB1* (1236G > A rs1128503/2677C > A/T rs2032582/3435G > A rs1045642) in our patients, we found that the haplotype AA/AA/AA was a protective factor against relapse 0.632 OR (CI 95% 0.495–0.805, *p* = .040). However, the same genotypes were a risk factor for death after HSCT 13.73 OR (CI 95% 1.94–97.17, *p* = .009). On the other hand, *ABCB1* (1236G > A rs1128503/2677C > A/T rs2032582/3435G > A rs1045642) combined genotypes GA/CA/GA was a risk factor for relapse 4.3 OR (CI 95% 1.09–17.10, *p* = .037) in our cohort. In a study of adult AML patients, *ABCB1* (1236G > A rs1128503/2677C > A/T rs2032582/3435G > A rs1045642) AA/AA/AA genotypes analyzed with other SNV genes showed an increased risk for nephrotoxicity and liver toxicity.^35^

In a previous study, *DCK* gene (‐360C > T, rs377182313) and (‐201C > A, rs2306744) were evaluated among AML adult patients. They found that patients with (‐360C > T, rs377182313) CG and (‐201C > A, rs2306744) CT and (‐360C > T, rs377182313) GG and (‐201C > A, rs2306744) TT compound genotypes displayed a favorable response to chemotherapy and increased expression of dCK mRNA, whereas those with (‐360C > T, rs377182313) CC and (‐201C > A, rs2306744) CC tended to have a poor response and lower expression of mRNA (*p* = .025 and *p* = .0034, respectively).^22^ Although (‐360C > T, rs377182313) was not included, no association was found for (‐201C > A, rs2306744) in this cohort.

Previously, *CDA* (79A > C, rs2072671) CC and *DCK* (‐201C > A, rs2306744) CC genotypes were associated with increased risk of death and *DCK* (‐201C > A, rs2306744) CC genotype as a risk factor for toxicity grades >3 in a cohort of 27 Mexican patients.^36^ We did not find any of these associations, instead, *DCK* (‐201C > A, rs2306744) GA genotype was found to be a protective factor for toxicity after HSCT.

In another study, the *CDA* (79A > C, rs2072671) CC genotype was associated with increased cytotoxicity when exposed to cytarabine in AML patients as compared with AA genotype.^3^ There was no association between this genotype and any toxicity in our cohort. Megias‐Vericat et al, found that *CDA* (79A > C, rs2072671) AC genotype was associated with overall survival at 5 years 2.2 OR (CI 95% 1.2–4.5, *p* = .015), event free survival 1.9 OR (CI 95% 1.01–3.4, *p* = .045) and relapse free survival 9.1 OR (CI 95% 1.2–68.6, *p* = .032).^37^ There was no association between this genotype and better clinical outcomes in our cohort. Also, *CDA* (‐451C > T, rs532545) TT genotype was associated with increased cytotoxicity when exposed to cytarabine in AML patients as compared to CC genotype.^24^ In our cohort, we found that *CDA* (‐451C > T, rs532545) CC genotype was a protective factor against colitis 0.2 OR (CI 95% 0.048–0.828, *p* = .019). Parmar et al. reported *CDA* 79A > C rs2072671 AC + CC genotypes associated with increased drug toxicity when treated with cytarabine as compared to AA genotype in an in vitro assay on healthy volunteers.^25^ In our cohort, no effects were found for any combined *CDA* genotypes.

In our cohort, no association was found between *ABCB1* (1236G > A rs1128503/2677C > A/T rs2032582/3435G > A rs1045642) AA/AA/AA genotypes and toxicity outcomes when combined with *CDA* (79A > C, rs2072671) or (‐451C > T, rs532545) and *DCK* (‐201C > A, rs2306744). However, *ABCB1* (1236G > A rs1128503/2677C > A/T rs2032582/3435G > A rs1045642) GG/CC/GG genotype in addition to *CDA* (79A > C, rs2072671) CA or *CDA* (‐451C > T, rs532545) CT genotypes showed a higher risk for MRD >0.1% at the end of the first cycle of induction, an important prognostic factor for overall survival and event‐free survival.^38^, ^39^

Previously, *GSTT1* null genotype was associated with an increased rate of early death after the initiation of chemotherapy in Japanese AML patients treated with cytarabine, mercaptopurine, prednisone, and daunorubicin.^38^ In our cohort, no association between *GSTT1* and *GSTM1* and toxicity effects were found, probably due to sample size (data not shown).

There is scanty information published on pharmacogenetic associations in pediatric AML evaluating *CDA, DCK*, and *ABCB1* genes. In our cohort, the majority of genotypic associations between these gene variants and toxicity or clinical outcomes were different from previous studies. However, most reports analyzing these gene variants have been reported mainly in adult patients, and in other admixed populations different from our cohort. Further studies are needed to evaluate the associations found here, since this is the first study made in colombian population, which is a highly structured population due to admixture between European derived, Amerindians, and African descent populations. For example, the reported genotype frequencies for *CDA* (79A > C, rs2072671) in a Mexican cohort^36^ are quite different to those reported for a Spanish cohort^37^ and our sample. For *DCK* (‐201C > A, rs2306744), our genotype frequencies were similar to those reported previously,^37^ but quite different from those reported in the Mexican cohort.^36^

## 5 CONCLUSIONS

This is the first study of AML pharmacogenetics in Colombia, a country with a highly admix population structure. Here, we have used a SNaPShot™ assay to simultaneously analyze SNV in the *CDA, DCK*, and *ABCB1* genes. With this assay, we can run one sample at a time at very low cost, and results could be obtained at the same time as the cytogenetics studies. Although some genetic associations were found, the low number of pediatric AML cases analyzed could be a limitation and further studies will be required to validate the associations found in an independent cohort. Pharmacogenomics might be useful as a future tool for patient stratification for treatment with different chemotherapy regimens.

## AUTHOR CONTRIBUTIONS

**Luz K. Yunis:** Conceptualization (equal); data curation (equal); formal analysis (equal); funding acquisition (equal); investigation (equal); methodology (equal); writing – original draft (equal); writing – review and editing (equal). **Adriana Linares‐Ballesteros:** Conceptualization (lead); data curation (lead); formal analysis (lead); investigation (lead); methodology (lead); supervision (lead); writing – original draft (lead); writing – review and editing (lead). **Nelson Aponte:** Formal analysis (supporting); methodology (supporting). **Gisela Barros:** Data curation (supporting); investigation (supporting). **Johnny García:** Data curation (supporting); investigation (supporting). **Laura Niño:** Data curation (supporting); investigation (supporting). **Gloria Uribe:** Methodology (supporting). **Edna Quintero:** Methodology (supporting). **Juan J. Yunis:** Conceptualization (lead); data curation (lead); formal analysis (lead); funding acquisition (lead); investigation (lead); methodology (lead); supervision (lead); writing – original draft (lead); writing – review and editing (lead).

## CONFLICT OF INTEREST

The authors have stated explicitly that there are no conflicts of interest in connection with this article.

## ETHICS STATEMENT

Informed consent was obtained from each patient or guardian. This protocol was approved by the institutional ethics committee of the participant institutions. This project was approved by the ethics committee of each institution, CEI 125‐18 and 243‐1 (HOMI Fundación Hospital Pediátrico La Misericordia and Clínica Infantil Colsubsidio Bogotá D.C., Colombia, respectively) and the ethics committee of Universidad Nacional de Colombia (007‐091‐18).

## Supporting information


**Supplementary Figure S1.** Schematic representation of genes analyzed in the anthracyclines (idarrubicine) and cytarabine pathway.

**Supplemental Table S1** *ABCB1, CDA, DCK, GSTT1* and *GSTM1* genotypes of pediatric AML patients.

**Supplemental Table S2** Toxicity definitions according to CTCAE version 5.

## Data Availability

The data that support the findings of this study are not openly available and are available from the corresponding author upon reasonable request.
