# Supplementary material for: Pharmacogenetics of ABCB1 , CDA , DCK , GSTT1 , GSTM1 and outcomes in a cohort of pediatric acute myeloid leukemia patients from Colombia
Source: Cancer Rep (Hoboken). 2022 Oct 31;6(3):e1744. doi: 10.1002/cnr2.1744 (PMC10026301; doi:10.1002/cnr2.1744)
Supplement: Supplementary file 1 — Supplementary Figure S1. Schematic representation of genes analyzed in the anthracyclines (idarrubicine) and cytarabine pathway. [file CNR2-6-e1744-s002.docx]

**Supplementary figure 1. Schematic representation of genes analyzed in the anthracyclines (idarrubicine) and cytarabine pathway.**


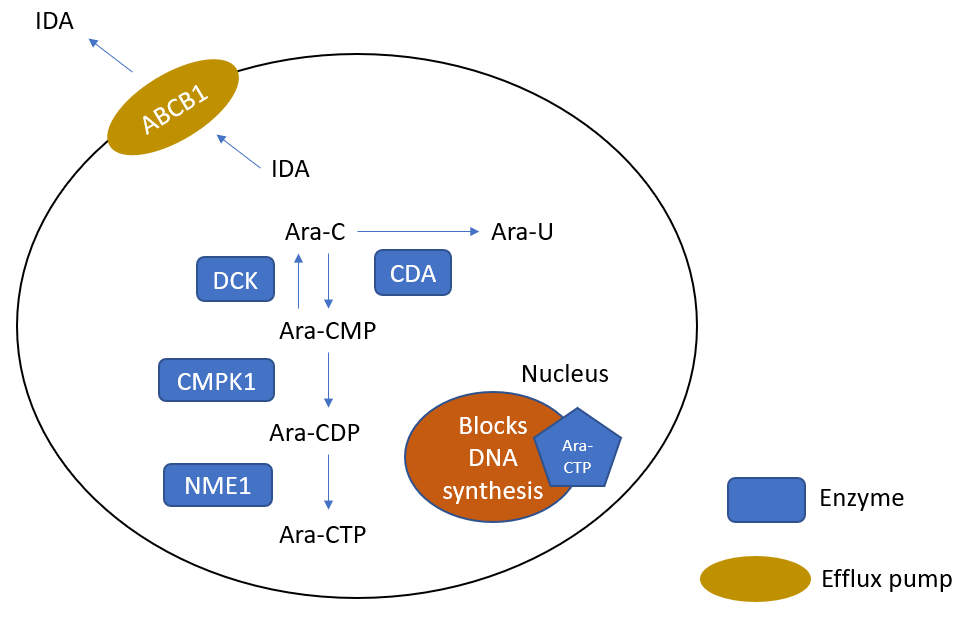


IDA: Idarrubicine; Ara-C: Cytarabine; Ara-CMP: Cytaranine monophosphate; Ara-CDP: Cytaranine diphosphate; Ara-CTP: Cytaranine triphosphate; Ara-U: Uracil arabinoside.
