# Supplementary material for: Pharmacogenetics of ABCB1 , CDA , DCK , GSTT1 , GSTM1 and outcomes in a cohort of pediatric acute myeloid leukemia patients from Colombia
Source: Cancer Rep (Hoboken). 2022 Oct 31;6(3):e1744. doi: 10.1002/cnr2.1744 (PMC10026301; doi:10.1002/cnr2.1744)
Supplement: Supplementary file 2 — Supplemental Table S1 ABCB1, CDA, DCK, GSTT1 and GSTM1 genotypes of pediatric AML patients. [file CNR2-6-e1744-s001.docx]

**Supplemental table 1** *ABCB1, CDA, DCK, GSTT1* and *GSTM1* genotypes of pediatric AML patients.

|  | ***ABCB1* 1236** | ***ABCB1* 2677** | ***ABCB1* 3435** | ***CDA* -451** | ***CDA* 79** | ***DCK* -201** | ***GSTT1*** | ***GSTM1*** |
| --- | --- | --- | --- | --- | --- | --- | --- | --- |
| LMA01 | GA | CA | GA | CT | CA | GG | 1 | 2 |
| LMA02 | GA | CA | GA | CC | AA | GG | 2 | 1 |
| LMA03 | GG | CC | GG | CT | CA | GG | 1 | 1 |
| LMA04 | GA | CA | GA | CT | CA | GG | 1 | 2 |
| LMA05 | GG | CC | GG | TT | CA | GG | 1 | 1 |
| LMA06 | GG | CT | GG | CT | CA | GG | 2 | 1 |
| LMA07 | GA | CA | AA | CC | AA | GG | 1 | 1 |
| LMA08 | AA | AA | AA | CC | AA | GG | 1 | 2 |
| LMA09 | AA | AA | AA | CC | AA | GG | 1 | 2 |
| LMA10 | AA | AA | GA | CT | CA | GG | 1 | 1 |
| LMA11 | GA | CA | GA | CC | AA | GG | 1 | 2 |
| LMA12 | AA | CA | GA | CT | CA | GG | 1 | 2 |
| LMA14 | AA | AA | AA | CC | AA | GG | 1 | 2 |
| LMA15 | GA | CA | AA | CT | CA | GG | 2 | 2 |
| LMA16 | GG | CC | GG | CT | CA | GA | 1 | 1 |
| LMA17 | GG | CC | GG | CT | CA | GG | 1 | 1 |
| LMA18 | GG | CC | GA | CT | CA | GG | 1 | 2 |
| LMA19 | GA | CA | GA | CC | AA | GG | 2 | 2 |
| LMA20 | GG | CC | GA | CT | CA | GG | 1 | 1 |
| LMA21 | GA | CA | GA | CT | CA | GG | 1 | 1 |
| LMA22 | AA | AA | AA | TT | CA | GG | 1 | 1 |
| LMA23 | AA | AA | AA | CT | CA | GG | 1 | 1 |
| LMA24 | GA | CA | GA | CT | CA | GG | 1 | 1 |
| LMA25 | GA | CA | GA | CT | CA | GG | 1 | 1 |
| LMA26 | GA | CA | GA | CC | AA | GG | 1 | 1 |
| LMA27 | GG | CC | GG | CC | AA | GG | 1 | 2 |
| LMA28 | GA | CA | GA | CT | CA | GG | 1 | 1 |
| LMA29 | GG | CC | GA | TT | CA | GA | 1 | 2 |
| LMA30 | GG | CA | GG | CT | CA | GG | 1 | 1 |
| LMA31 | AA | AA | AA | CC | AA | AA | 2 | 2 |
| LMA32 | GA | CA | GG | CT | CA | GG | 1 | 1 |
| LMA33 | GA | CA | GA | CT | CA | GA | 1 | 1 |
| LMA34 | GG | CA | GG | CT | AA | GG | 1 | 1 |
| LMA35 | AA | CA | GA | CC | AA | GG | 1 | 1 |
| LMA36 | GA | CA | GA | CC | AA | GG | 1 | 2 |
| LMA37 | AA | AA | AA | CT | CA | GG | 1 | 1 |
| LMA38 | AA | AA | GA | CT | CA | GA | 1 | 1 |
| LMA39 | GA | CA | GG | CT | AA | GG | 1 | 1 |
| LMA40 | AA | CA | GA | CT | CA | GG | 1 | 2 |
| LMA41 | AA | AA | AA | CC | AA | GG | 1 | 2 |
| LMA42 | GG | CC | GG | CC | AA | GA | 1 | 1 |
| LMA43 | GA | CA | GA | CC | CA | GG | 1 | 1 |
| LMA44 | GA | CA | GA | CC | AA | GG | 1 | 1 |
| LMA45 | GA | CA | GA | CT | CA | GG | 1 | 1 |
| LMA46 | GA | CA | GA | CC | AA | GG | 1 | 1 |
| LMA47 | AA | AA | AA | TT | CA | GG | 1 | 2 |
| LMA48 | GG | CC | GG | CC | AA | GG | 1 | 1 |
| LMA49 | GG | CC | AA | CT | CA | GG | 1 | 1 |
| LMA50 | AA | CA | GA | CT | CA | GG | 1 | 2 |
| LMA51 | AA | AA | GA | CT | CA | F | 1 | 2 |
| LMA52 | AA | AA | GA | CT | CA | GG | 1 | 1 |

F: Failed; 1: present; 2: absent.
