# Supplementary material for: Pharmacogenetics of ABCB1 , CDA , DCK , GSTT1 , GSTM1 and outcomes in a cohort of pediatric acute myeloid leukemia patients from Colombia
Source: Cancer Rep (Hoboken). 2022 Oct 31;6(3):e1744. doi: 10.1002/cnr2.1744 (PMC10026301; doi:10.1002/cnr2.1744)
Supplement: Supplementary file 3 — Supplemental Table S2 Toxicity definitions according to CTCAE version 5. [file CNR2-6-e1744-s003.docx]

**Toxicity definitions according to CTCAE version 5.**

| **CTCAE Term** | **Definition** | **Grade 1** | **Grade 2** | **Grade 3** | **Grade 4** |
| --- | --- | --- | --- | --- | --- |
| Oral mucositis | A disorder characterized by ulceration or inflammation of the oral mucosal. | Asymptomatic or mild symptoms; intervention not indicated | Moderate pain or ulcer that does not interfere with oral intake; modified diet indicated | Severe pain; interfering with oral intake | Life-threatening consequences; urgent intervention indicated |
| Colitis | A disorder characterized by inflammation of the colon. | Asymptomatic; clinical or diagnostic observations only; intervention not indicated | Abdominal pain; mucus or blood in stool | Severe abdominal pain; peritoneal signs | Life-threatening consequences; urgent intervention indicated |
| Transaminitis-  increased Alanine aminotransferase | A finding based on laboratory test results that indicate an increase in the level of alanine aminotransferase (ALT or SGPT) in the blood specimen. | >ULN - 3.0 x ULN if baseline was normal; 1.5 - 3.0 x baseline if baseline was abnormal | >3.0 - 5.0 x ULN if baseline was normal; >3.0 - 5.0 x baseline if baseline was abnormal | >5.0 - 20.0 x ULN if baseline was normal; >5.0 - 20.0 x baseline if baseline was abnormal | >20.0 x ULN if baseline was normal; >20.0 x baseline if baseline was abnormal |
| Transaminitis-  increased Aspartate aminotransferase | A finding based on laboratory test results that indicate an increase in the level of aspartate aminotransferase (AST or SGOT) in a blood specimen. | >ULN - 3.0 x ULN if baseline was normal; 1.5 - 3.0 x baseline if baseline was abnormal | >3.0 - 5.0 x ULN if baseline was normal; >3.0 - 5.0 x baseline if baseline was abnormal | >5.0 - 20.0 x ULN if baseline was normal; >5.0 - 20.0 x baseline if baseline was abnormal | >20.0 x ULN if baseline was normal; >20.0 x baseline if baseline was abnormal |
| Cardiotoxicity- Left ventricular systolic dysfunction | A disorder characterized by failure of the left ventricle to produce adequate output. | - | - | Symptomatic due to drop in ejection fraction responsive to intervention | Refractory or poorly controlled heart failure due to drop in ejection fraction; intervention such as ventricular assist device, intravenous vasopressor support, or heart transplant indicated |
| Cardiotoxicity- Heart failure | A disorder characterized by the inability of the heart to pump blood at an adequate volume to meet tissue metabolic requirements, or, the ability to do so only at an elevation in the filling pressure. | Asymptomatic with laboratory (e.g., BNP [B-Natriuretic Peptide ]) or cardiac imaging abnormalities | Symptoms with moderate activity or exertion | Symptoms at rest or with minimal activity or exertion; hospitalization; new onset of symptoms | Life-threatening consequences; urgent intervention indicated (e.g., continuous IV therapy or mechanical hemodynamic support) |
| Cardiotoxicity- Conduction disorder | A disorder characterized by pathological irregularities in the cardiac conduction system. | Mild symptoms; intervention not indicated | Non-urgent medical intervention indicated | Symptomatic, urgent intervention indicated | Life-threatening consequences |
